# PLGA nanoparticles loaded with Gallic acid- a constituent of *Leea indica* against *Acanthamoeba triangularis*

**DOI:** 10.1038/s41598-020-65728-0

**Published:** 2020-06-02

**Authors:** Tooba Mahboob, Muhammad Nawaz, Maria de Lourdes Pereira, Tan Tian-Chye, Chandramathi Samudi, Shamala Devi Sekaran, Christophe Wiart, Veeranoot Nissapatorn

**Affiliations:** 1 Department of Medical Microbiology, Faculty of Medicine, University of Malaya, 50603 Kuala Lumpur, Malaysia; 2 Department of Parasitology, Faculty of Medicine, University of Malaya, 50603 Kuala Lumpur, Malaysia; 3 Department of Nano-Medicine Research, Institute for Research and Medical Consultations (IRMC), Imam Abdulrahman Bin Faisal University, P.O. Box 1982, 31441 Dammam, Saudi Arabia; 4 Department of Medical Sciences & CICECO-Aveiro Institute of Materials, University of Aveiro, 3810-193 Aveiro, Portugal; 5 Faculty of Medicine, MAHSA University, Saujana Putra Campus, Selangor, Malaysia; 6 School of Pharmacy, Nottingham University Malaysia Campus, 43500 Semenyih, Selangor, Kuala Lumpur, Malaysia; 7 School of Allied Health Sciences, Southeast Asia Water Team (SEA Water Team), and World Union for Herbal Drug Discovery (WUHeDD), Walailak University, 80161 Nakhon Si, Thammarat Thailand; 8 Research Excellence Center for Innovation and Health Products (RECIHP), Walailak University, 80161 Nakhon Si, Thammarat Thailand

**Keywords:** Drug delivery, Infection

## Abstract

*Acanthamoeba*, a genus that contains at least 24 species of free-living protozoa, is ubiquitous in nature. Successful treatment of *Acanthamoeba* infections is always very difficult and not always effective. More effective drugs must be developed, and medicinal plants may have a pivotal part in the future of drug discovery. Our research focused on investigating the *in vitro* anti- acanthamoebic potential of *Leea indica* and its constituent gallic acid in different concentrations. Water and butanol fractions exhibited significant amoebicidal activity against trophozoites and cysts. Gallic acid (100 µg/mL) revealed 83% inhibition of trophozoites and 69% inhibition of cysts. The butanol fraction induced apoptosis in trophozoites, which was observed using tunnel assay. The cytotoxicity of the fractions and gallic acid was investigated against MRC-5 and no adverse effects were observed. Gallic acid was successfully loaded within poly (D,L-lactide-co-glycolide) (PLGA) nanoparticles with 82.86% encapsulation efficiency, while gallic acid showed 98.24% *in vitro* release at 48 hours. Moreover, the gallic acid encapsulated in the PLGA nanoparticles exhibited 90% inhibition against trophozoites. In addition, gallic acid encapsulated nanoparticles showed reduced cytotoxicity towards MRC-5 compared to gallic acid, which evidenced that natural product nanoencapsulation in polymeric nanoparticles could play an important role in the delivery of natural products.

## Introduction

*Leea indica* (Burm. f.) Merr. (Family-*Leeaceae*), commonly known as kur jiwa, arengi or achila gaeh, is widespread in tropical and subtropical regions, for example, Malaysia, China, Thailand, India and Bangladesh^1^. Several known chemical compounds have been isolated from the leaves of *Leea indica*, which includes phthalic acid, ursolic acid, gallic acid, β-sitosterol, palmitic acid, farnesol, lupeol and 1-eicosanol. Apart from that, Srinivasan *et al*. identified other compounds such as lycopersen, heptadecane, isooctyl phthalate, butyl-2-phthalate, butyl-2-ethylhexyl phthalate, di-n-butyl phthalate, butyl gallate, triterpenes and sterol from the methanol extract^2^. Among these isolated compounds, di-n-butyl phthalate exhibited antibacterial and antifungal properties^3^, while butyl gallate was an antioxidant^4^. Interestingly, gallic acid is the one that has been reported to have several biological activities, mainly antioxidant, anti-tyrosinase, antimicrobial, anti-inflammatory, anticancer and neuro-protective activities^5^. Gallic acid was isolated from several medicinal plants, including the butanol fraction of *Crassula ovata* (Mill.) Druce leaves by Tombozara *et al*.^6^. More recently, through LC-MS/MS, some phytochemicals have been identified in the leaves of *Leea indica*, such as phenols, flavonoids, benzoic acid derivatives, dihydrochalcones, coumarins, catechins, oxylipins and megastigmans^7^. It is a green shrub with a maximum growth of 2–3 m in height, with firm and soft woody shoots^2^. *L. indica* is used to treat cancer, diabetes, heart disease, skin problems, such as skin rashes and allergic reactions^8,9^, and various diseases, such as fever and dizziness, as well as diarrhea and chronic dysentery^10^, an inflammatory disease of the lower intestinal tract, commonly caused by bacterial, parasitic, or protozoal infection^11,12^. Leaves are generally expended crude or taken in blended form from fresh leaves^13^. There are several traditional uses of *L. indica* as herbal medicines by the tribes of Bangladesh. The leaves are used for obstetric diseases, body pain, diabetes, vertigo and birth control^14^. The root of *L. indica* is used as an antidysenteric, antidiarrheal, antiplasmodic and also to treat heart disease and cancer. It is reported that the whole plant is used for body aches, headaches and skin complaints. *L. indica* is believed to have therapeutic activities to expel wind and eliminate dampness (Chinese: 驅風祛濕, Dampness) in traditional Chinese medicine, as well as clearing internal heat and poison (Chinese: 清熱解毒, Detoxification). Here “poison” is a term in traditional Chinese medicine that refers to the body´s infectious factor^15^. Recently, Siew (2019) using several cancer cell lines, demonstrated the great effectiveness of the methanol extract of *L. indica*^16^. Overall, *L. indica* is reported to have high medical values. It is a convenient, inexpensive and potent antibiotic with minimal toxicity. However, its anti-amoebic potential needs further investigation.

Free-living amoebas (FLA) are universal protozoa, found in soil, air and water. They are part of natural and artificial ecosystem. One of the main reasons for the expansion of these pathogenic amoebas is the transformation of plants into natural specialties by Wastewater Treatment Plants (WTPs). These ecological niches are suitable for the colonization of FLAs and establish their territory, since they feed on microorganisms, mainly bacteria. Forms of organic cleaning are not intended to evacuate microbiological defilement, although they help to decrease some microbial populations. Currently, only a few genera and types of FLAs have been portrayed as pathogenic, but each represents a risk as a supply of pathogens. Their cystic stage provides protection against unfavorable conditions and regular disinfectants, allowing them to resist forms of water refinement and colonize in treated water structures. In previous examinations, pathogenic *Acanthamoeba* was isolated from different water sources which overwhelmingly incorporate channel water, swimming pool, rain storage, ponds and lakes^17–19^.

The mortality or morbidity rate associated with *Acanthamoeba* infections varies according to the type of disease. As in amoebic keratitis, the disease does not turn into systemic infection or death. However, other factors, including progressive visual loss, abscess formation, cataracts, scleritis, glaucoma and ulceration, can lead to complications in keratitis infection^20^. Delay in therapy, use of steroids, and development of extracoronal manifestations are common features that contribute for worse ocular prognosis. Current drugs have limited efficacy, except for use in early stages^20^. Granulomatous amoebic encephalitis (GAE) has a high mortality rate of around 100%. Apart from that, disseminated infections without central nervous system (CNS) involvement account for high mortality rates, although still lower than GAE^21^. Treatments against *Acanthamoeba* infections, including natural products and various chemotherapeutic agents, have been reported in *in vitro* studies for potential amoebicidal activity, but the results remain controversial. Adequate information is still lacking as to whether these agents are effective in patients with granulomatous amoebic encephalitis (GAE)^22–24^. Combined therapy has been found to be more effective than a single drug used in human infections. No single drug is active against the cyst and trophozoite stages of *Acanthamoeba* infections, which can result in spread to the lungs, skin, central nervous system (CNS), and other organs, depending on the host´s immune status. Therefore, increasing awareness of *Acanthamoeba* as a potential source of infections is critical, as it is important for early diagnosis and treatment. Treatments with pentamidine, ketoconazole, fluconazole, itraconazole, metronidazole and amphotericin B do not treat lesions in immunosuppressed patients with sinusitis and skin lesions^25^. Based on the literature review, much research is being conducted to explore new effective, less toxic and low-cost therapeutic agents for the treatment of *Acanthamoeba* infections. However, the emergence of resistance to common antimicrobials agents has been remarkably observed in recent years in the treatment of amoebic keratitis (AK)^26–28^. The apparent cause of this resistance is the presence of the cyst cell wall as a physical barrier, and not as a result of the dormant cyst itself. Lazuana and colleagues (2019) have shown that cellulase has the potential to degrade the cyst wall of *Acanthamoeba* sp. cysts^29^.

Nanotechnology is a branch of applied science and technology which deals with the development of devices and dosage forms in the range of 1 to 100 nm. This new technology has emerged as a new approach to investigate new paths in which traditional methodologies have failed to impact the diagnosis, prevention, and treatment of various diseases^30^. The delivery of innumerable therapeutic agents or active compounds at the desired site of action can be adequately enhanced by nanometer-sized entities, maintaining the activity of the loaded drug^31–35^. Specifically, nanotechnology entities can be used to overcome problems such as short half-life, low solubility and reduce toxicity, which will help to deliver conventional natural products more efficiently.

Thus, this study proposes to assess the anti-amoebic potential of several fractions of *Leea indica* and its constituent gallic acid. In this study, gallic acid was encapsulated in poly (D,L-lactide-co-glycolide) PLGA nanoparticles to increase efficiency with minimal cytotoxic effects. The current study was conducted against pathogenic *Acanthamoeba* as an effort to develop new, more effective therapeutic agents with less cytotoxicity.

## Results

### Amoebicidal assay

#### Trophozoites

**Figure 1:**
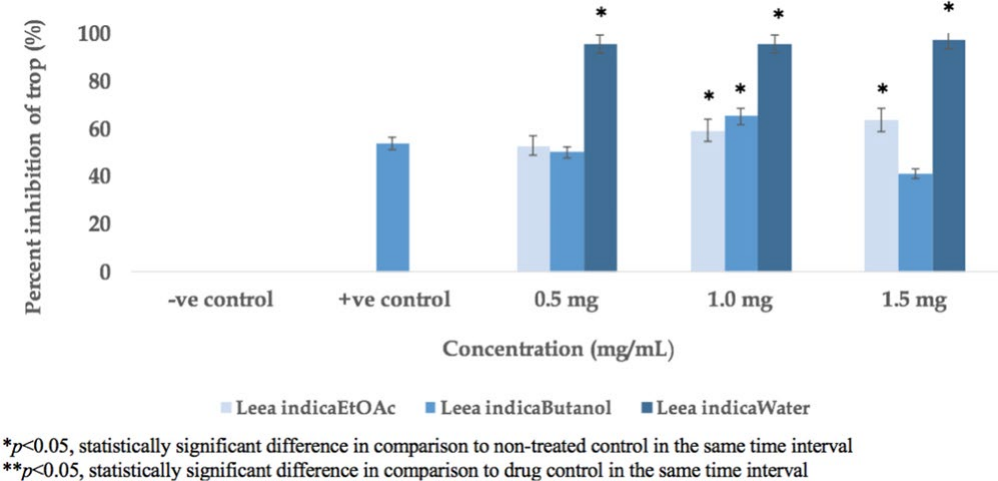
Trophocidal Activity of *Leea indica* Fractions.

All fractions of *Leea indica* showed potent trophocidal activity at three different concentrations ranging from 0.5 to 1.5 mg/mL. However, the aqueous fraction of *Leea indica* showed more than 80% of trophocidal activity at all concentrations (0.5–1.0 mg/mL), considered significant compared to standard chlorhexidine. The *Leea indica* butanol fraction exhibited 60% trophocidal activity at 1.0 mg/mL, with the maximum decrease in amoebicidal activity shown by ethyl acetate fraction of *Leea indica* (60% at 1.5 mg/mL) (Fig. 1).

#### Cysts

**Figure 2:**
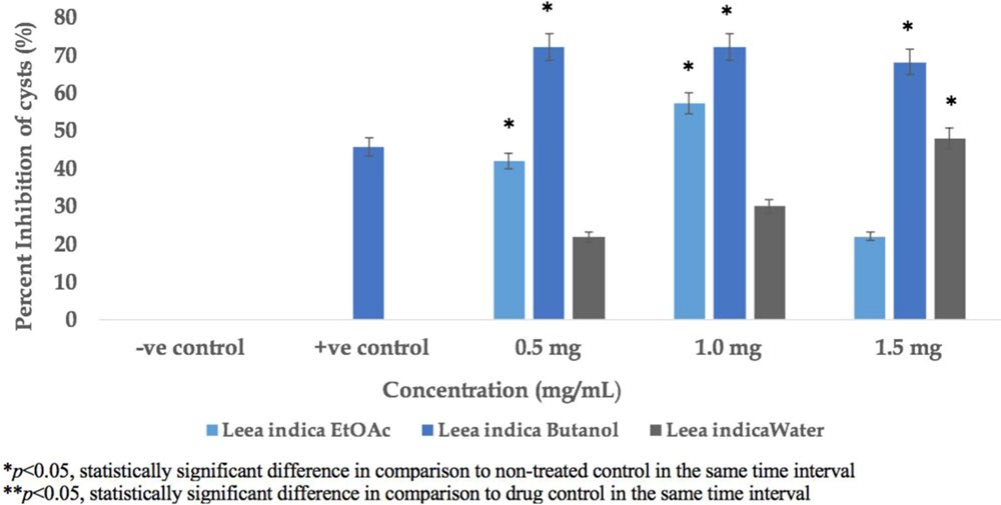
Cysticidal Activity of *Leea indica* Fractions.

The cysticidal potential of the water, butanol and ethyl acetate fractions was evaluated at three different concentrations in the range of 0.5 to 1.5 mg/mL compared to commercially available chlorhexidine. The butanol fraction of *Leea indica* exhibited the highest cysticidal activity by approximately 70% in the three concentrations. This cysticidal potential of butanol fraction of *Leea indica* was found to be significant compared to standard chlorhexidine. The ethyl acetate fraction of *Leea indica* indicated 55% inhibition of cysts at a concentration of 1.0 mg/mL, while water fraction of *Leea indica* showed the least inhibition in *Acanthamoeba* cysts (45% inhibited cysts at 1.5 mg/mL) (Fig. 2).

### Standardization and amoebicidal activity

**Figure 3:**
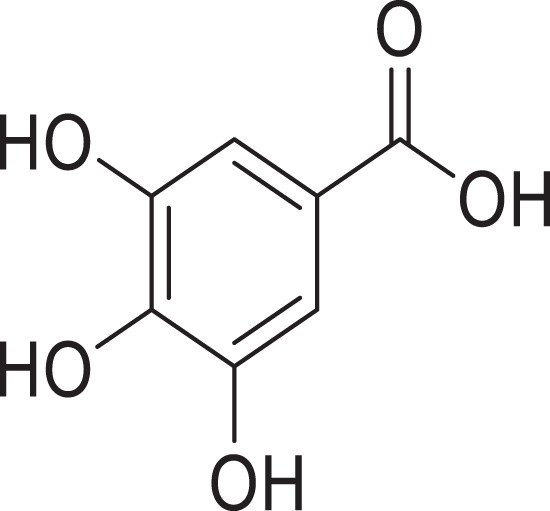
Chemical structure of gallic acid.

**Figure 4:**
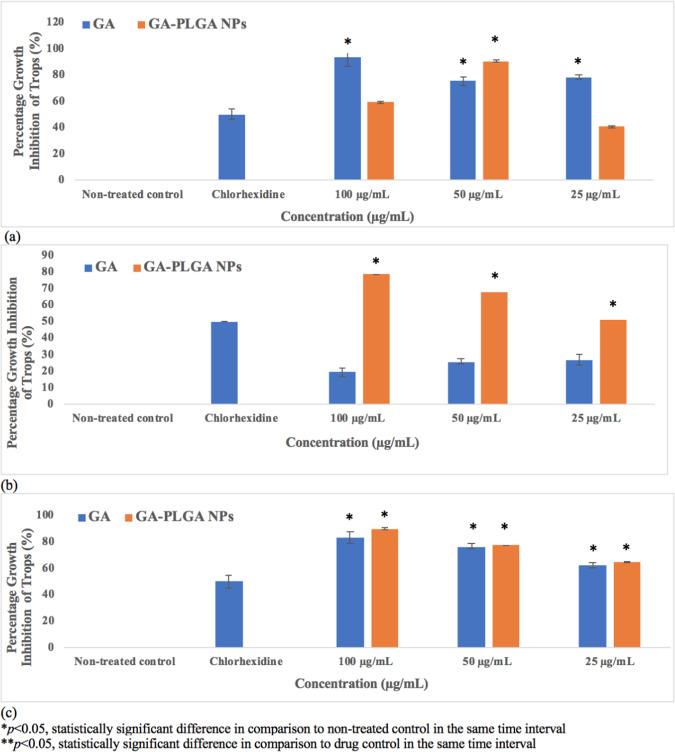
Trophocidal Activity of Gallic Acid (GA) and Nano-GA-PLGA after 24 hours (**a**), 48 hours (**b**), 72 hours (**c**).

**Figure 5:**
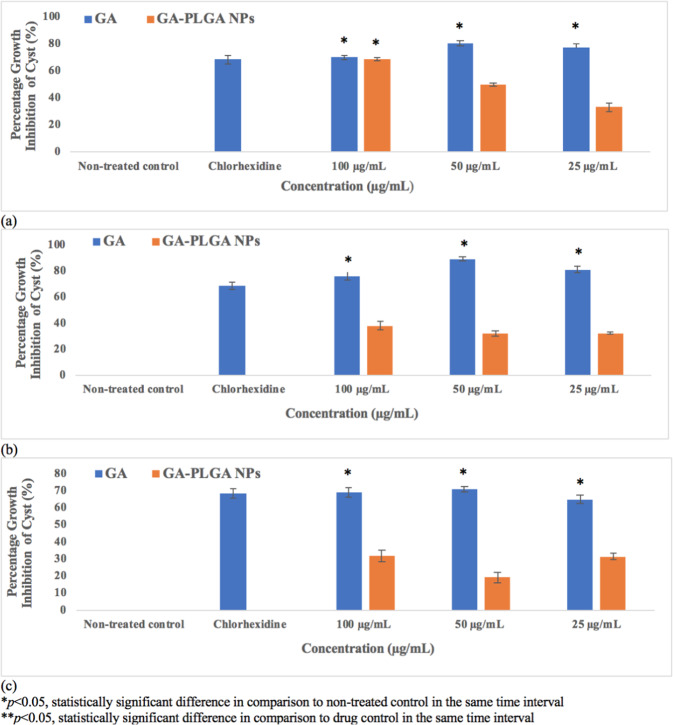
Cysticidal Activity of Gallic Acid (GA) and Nano-GA-PLGA after 24 hours (**a**), 48 hours (**b**), 72 hours (**c**).

The main constituent of *Leea indica* was confirmed by standardization with standard gallic acid (Fig. 3). Gallic acid was eluted at the 32-minute retention time of the *Leea indica* butanol fraction. Its constituent, gallic acid was evaluated for its activity against *Acanthamoeba* (trophozoites and cysts) in the concentration ranges from 25 to 100 µg/mL. Gallic acid showed inhibition of 83, 76.4 and 62.3% at concentrations of 100 (587.8 µM), 50 (293.9 µM) and 25 µg/mL (146.9 µM), respectively, 72 hours later against trophozoites (Fig. 4). As a result, the cysticidal activity of gallic acid was 69.0, 71.1 and 64.8% at 100 (587.8 µM), 50 (293.9 µM) and 25 µg/mL (146.9 µM), respectively (Fig. 5).

### Size and morphology of Nano-GA-PLGA

**Figure 6:**
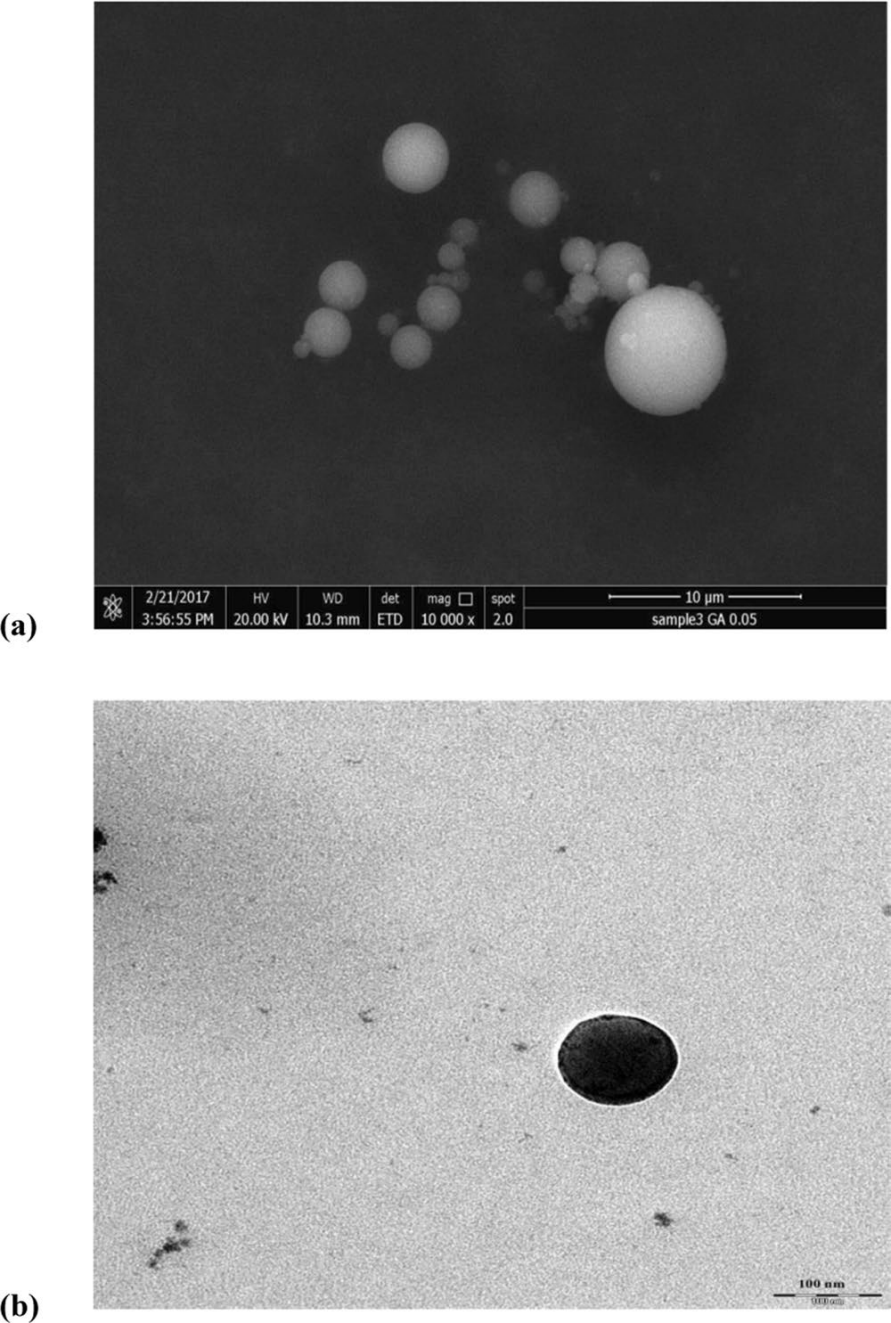
(**a**) Scanning Electron Microscopy (SEM) of synthesized Nano-GA-PLGA. (**b**) Transmission Electron Microscopy (TEM) of synthesized Nano-GA-PLGA.

The size and morphological examination of the gallic acid encapsulated in PLGA nanoparticles were performed by SEM and TEM analysis. SEM and TEM revealed that PLGA nanoparticles loaded with gallic acid were sphere-shaped and approximately 100 nm in size. However, few synthesized nano-GA-PLGA have sheet-like morphological characteristics and 100 nm in size (approx.). All particles showed a smooth surface with wide dispersion of size and high capacity to form clusters (Fig. 6a,b). Probably, the aggregation of gallic acid nanoparticles may be due to the non-ionic surfactant used in the synthesis of nanoparticles, polyvinyl alcohol (PVA), which may result in insufficient steric stabilization. Moreover, the matrix is created by the bonding of hydrophobic polyvinyl alcohol groups with the PLGA chain, while hydrophilic polyvinyl alcohol groups face the water phase.

### Percent encapsulation and percent release

The spectrophotometer (SpectraMax, M3, Molecular Devices, Washington DC, USA) was used to confirm the encapsulation of gallic acid inside the PLGA nanoparticles. The absorbance was measured at 230 nm to detect free gallic acid and a standard curve was constructed to determine the concentration of gallic acid. Proficiency in trapping gallic acid was expressed as the percentage of active substance (gallic acid) loaded in the PLGA nanoparticles reported in the initial amount of gallic acid used for nanoparticles synthesis. Determinations were made in triplicates. The encapsulation efficiency allows for a specific amount of drug/compound encapsulated in the PLGA matrix. The encapsulation efficiency for gallic acid was 82.86%. Gallic acid showed maximum encapsulation efficiency in PLGA nanoparticles, after that no change in the encapsulation efficiency was observed, probably due to the saturation of gallic acid in the polymer dispersion^36^.

The *in vitro* release of gallic acid from nano-PLGA was studied in PBS at pH 7.4. The drug release was measured as a function of time. A maximum of 98.24% gallic acid was subsequently released after 48 hours (the release of drug from GA-PLGA nanoparticles within 24 hours was demonstrated in Fig. S3); no other releases were recorded after that. The release of the compound depends on several factors including temperature, type of nanoparticles, release medium and pH^36^. The burst release (21%) of GA from nano-PLGA was initially observed after 24 hours. The initial burst drug release may be due to the presence of gallic acid in the surface of PLGA NPs. The drug release was slow in the first 24 hours, as it had the polymer content and this has the impact of decelerating drug release as a result of the increase in the particle size and reduced surface area available for drug release. The percentage release of gallic acid was 98.24% from the GA- PLGA nanoparticles after 48 hours.

### Amoebicidal activity of Nano-GA-PLGA

Gallic acid was successfully loaded into nano-PLGA using the single emulsion method. The amoebicidal activity of the encapsulated nano-GA-PLGA was evaluated at different concentrations (25, 50 and 100 µg/mL) at 24, 48 and 72 hours, respectively. Nano-GA-PLGA reduced the growth of trophozoites by 90.7% after 24 hours at 50 µg/mL (Fig. 4). GA-PLGA nanoparticles showed significant trophocidal activity at 100 µg/mL after 48 and 72 hours, which was considered significant compared to standard chlorhexidine. After 72 hours, nano-GA-PLGA inhibited the viability of trophozoites by 64.9, 77.4 and 90.0% at 25, 50 and 100 µg/mL respectively. In contrast, nano-GA-PLGA showed 31.5 and 38% inhibition of cyst viability after 48 and 72 hours respectively (at 100 µg/mL) (Fig. 5).

### Cytotoxicity of GA and Nano-GA-PLGA on MRC-5 cells

**Figure 7:**
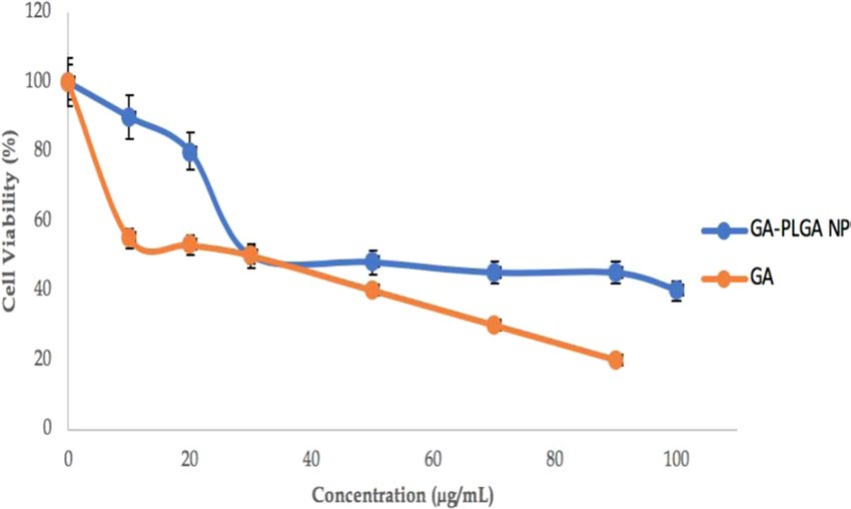
Cytotoxicity Tests of Gallic acid and Gallic acid NPs (Nano-GA- PLGA) against Lung epithelial cell line (MRC-5); IC_50_: GA- 10 µg/mL, IC_50_: Nano-GA- PLGA − 30 µg/mL.

The cytotoxicity assay was used to determine the cytotoxic effects of free gallic acid and gallic acid encapsulated in PLGA nanoparticles (Nano-GA-PLGA). Lung epithelial cells (MRC-5) were exposed to varying concentration of test compounds ranging from 10 to 100 µg/mL (Fig. 7). Before encapsulation in PLGA nanoparticles, the IC_50_ of gallic acid was 10 µg/mL. A distinct reduction in the cytotoxicity of gallic acid was observed after encapsulating gallic acid into PLGA nanoparticles with an IC_50_ of 30 µg/mL (Fig. 7). The reduction in cytotoxicity may be due to the encapsulation of gallic acid.

### Apoptotic effect of *Leea indica* fractions, GA and Nano-GA-PLGA

**Figure 8:**
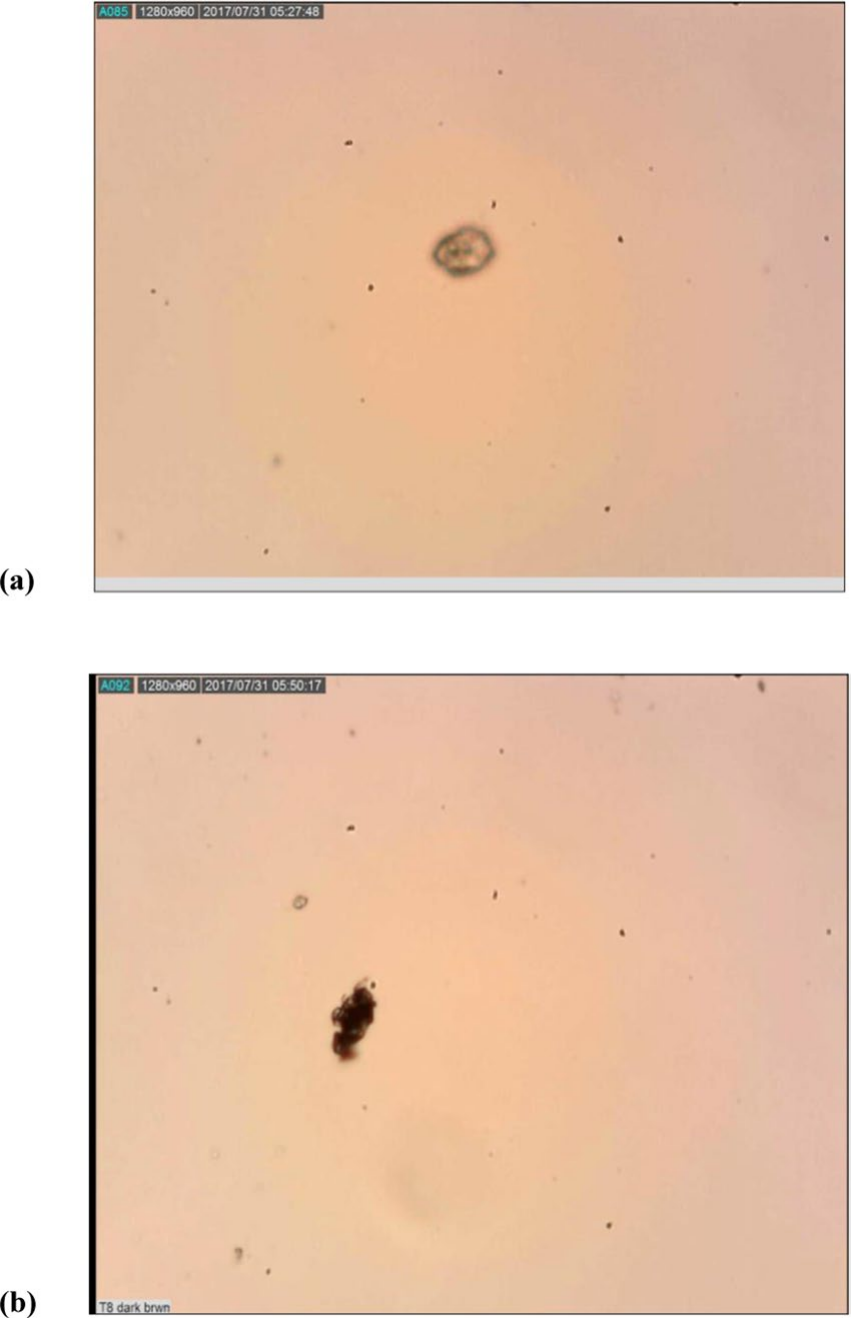
(**a**) Apoptotic Effect of *Leea indica* (Water and Ethyl acetate Fraction), GA, Nano-GA-PLGA (No DNA Fragmentation), (**b**) Apoptotic Effect of *Leea indica* Butanol Fraction (Showed DNA Fragmentation).

The programme cell death induced by fractions of *Leea indica* (butanol, water, and ethyl acetate), free gallic acid and gallic acid encapsulated in PLGA nanoparticles (Nano-GA-PLGA) was evaluated using the TUNEL assay (Fig. 8a,b). The concentrations at which *L. indica* fractions and GA inhibited 50% of *Acanthamoeba triangularis* were used in the assay. DNA fragmentation was observed in the *Leea indica* butanol fraction (Fig. 8b). Brown precipitate was observed as a result of terminal deoxynucleotidyl transferase (TdT) binding at the end of DNA fragments. It demonstrates that the apoptotic effect was caused by the butanol fraction of *Leea indica*.

## Discussion

Several infectious diseases continue to increase over time. Patients infected with *Acanthamoeba* have been reported in large numbers since the past few years. Similarly, strains of *Acanthamoeba* resistant to standard courses of current therapy are also being more frequently recognized^37^. Therefore, new therapeutic agents are urgently needed for effective, less toxic and affordable treatment. Natural products derived from plants and microorganisms are an important source for drug discovery - mainly for parasites, due to the long association between coexistence of parasite, human and traditional herbal medicines^38^.

In the present study, *L. indica* plant with three different fractions and its constituent, gallic acid were evaluated against pathogenic *Acanthamoeba* as an effort to develop new, more effective therapeutic agents with less cytotoxicity. *L. indica* is a small shrub found in abundance in Southeast Asia, China and Malaysia. Its leaves and roots have traditionally been used as antineoplastic, antidiabetic, antipyretic and antiallergic agents^8,39^. Its constituent, gallic acid, is a natural benzoic acid belongs to the class of simple phenols. It is abundant in green tea, oak bark, grapes, sumac and gallnuts^40,41^. In this study, we found that all fractions of *L. indica* showed promising potential against the cyst as well as trophozoites stages of *Acanthamoeba*, which had not been previously reported. In addition, gallic acid had strong anti-amoebic properties. Although gallic acid has been reported for several activities, such as antibacterial, antifungal and anti-inflammatory activity^42^. In recent years, new drug delivery techniques have been introduced in natural secondary metabolites in order to improve their bioavailability. Encapsulation has been tried in parasitic treatment as a carrier of antiparasitic agent to increase its effects and decrease cytotoxicity^43^. Polymeric nanoparticles have been successfully used in the treatment of mice infected with *Plamsodium yoelii* and inhibited the parasitic infection by 100%^44^. In our previous study *Pericampylus glaucus* and *Lonicera japonica* exhibited amoebicidal activity against cysts and trophozoites and anti-encystment activity. Secondary metabolites of *Pericampylus glaucus* (periglaucine A and betulinic acid), were successfully loaded and showed antiparasitic potential against pathogenic *Acanthamoeba*^45,46^. Chemoprevention through the use of natural products has developed as an influential strategy with the potential to combat infectious diseases. In this study, the butanol fraction of *L. indica* demonstrated a 90% trophocidal potential and almost 70% inhibition of the cysts (minimum concentration of 0.5 mg/mL). The best result and the eradication of 90% of the *Acanthamoeba* trophozoites were achieved by the polymeric nano-encapsulation of gallic acid (GA-PLGA nanoparticles). The process of programmed cell death was investigated in *Acanthamoeba* by *L. indica* fractions, gallic acid and synthesized GA-PLGA nanoparticles. *L. indica* butanol fraction induced apoptosis in *Acanthamoeba*, according to the previous study, where evidence of apoptosis in *Acanthamoeba* was reported^47^. However, no brown precipitate was observed in the cells by gallic acid and GA-PLGA nanoparticles, indicating that inhibitions in the growth of *Acanthamoeba* were not due to apoptosis. This observation suggests that there could be other non-apoptotic pathways, such as pyroptosis and necroptosis, which deserve further investigation.

The release of drug form nanoparticles is a relevant aspect in the development of nanoformulation. Some circumstances influence the rate of drug release from nanoparticles, such as diffusion though nanoparticles, erosion of the nanoparticle matrix, combined process of erosion/diffusion and desorption of adsorbed drug. Therefore, the release of the drug by the nano-polymer depends on the diffusion and biodegradation^48,49^. A higher percentage of drug release was observed in gallic acid encapsulated in PLGA nanoparticles (98.24%) after 48 hours.

The results of this study suggest that it is conceivable to control the release rate of gallic acid by adjusting the concentration of gallic acid and molecular parameters of PLGA. In contrast, the microsphere-based drug delivery system may have a higher percentage of burst release; therefore, a higher dose is generally needed^50^. The burst release can be controlled by reducing the polymer concentration. Drug release profiles are mainly affected by the evaporation temperature. Within a certain range of particle size, the rate of drug release is inversely proportional to the particle size and encapsulation efficiency, as drug release rate increases with the reduction in particle size^36^. Regarding cytotoxicity, we found that PLGA nanoparticles loaded with gallic acid showed a distinct reduction compared to unencapsulated gallic acid. Gallic acid, a strong antioxidant, despite having cell death-inducing effects on tumor cells, protects normal cells from the devastating effects of reactive oxygen species and therefore helps to extend the lifespan of peripheral blood lymphocytes (PBLs), which are one of the most important parts of the body’s defence system against various infections, including AIDS^5^.

## Materials and Methods

### Chemicals and Reagents

Poly (D,L-lactide-*co*-glycolide) (PLGA), Poly (vinyl alcohol**)** (PVA), quercetin, chlorhexidine, MTT (3-(4,5-dimethylthiazol-2-yl)-2,5-diphenyltetrazolium bromide), Diaminobenzidine (DAB), RPMI 1640 cell culture medium, Fetal bovine serum (FBS), Phosphate buffer saline (PBS), Trypan blue, Paraformaldehyde, Ethanol, Dimethyl sulfoxide (DMSO), dichloromethane, gallic acid and apoptosis detection kit were acquired from commercial companies.

### Plant collection

The leaves of *Leea indica* were freshly collected from the Penang Hill forest in Malaysia in February 2014 (322.24 g), and identified by Assoc. Prof. Dr Christophe Wiart, Faculty of Sciences, University of Nottingham, Malaysia Campus.

### Extraction and fractionation

Fresh leaves of *Leea indica* were soaked in liquid nitrogen (Lindle, Kuala Lumpur, Malaysia Batch no: UN1977) and crushed into small pieces. The crushed leaves (262.24 g) were then soaked in 5 L of 70% ethanol solution and 30% purified water for 7 days at room temperature (25 ± 2 °C) with occasional stirring. The extract was filtered through filter paper (Thermo Fisher Scientific, Winsford, United Kingdom). The whole filtrate was concentrated under reduced pressure at 55°C through a rotatory vacuum evaporator (R-200, Buchi, Switzerland) to produce concentrated crude ethanolic extract (13.7459 g dark greenish brown, yield 5.24% w/w). A volume of 120 mL of the concentrated crude ethanolic extract was used to extract even more with hexane (3 × 40 mL) to achieve hexane-soluble and hexane-insoluble fractions. The hexane-insoluble fraction was further extracted with 3 × 40 mL of ethyl acetate to obtain an extract soluble in ethyl acetate and a residue insoluble in ethyl acetate. The ethyl acetate-soluble portion was filtered through a decent amount of 97% pure anhydrous magnesium sulphate, (Acros Organics, Geel, Belgium), using filter paper (Thermo Fisher Scientific, Loughborough, United Kingdom) to remove any water contained therein. The residue insoluble in ethyl acetate was further partitioned between 3 × 40 mL of butan-1-ol to obtain the fraction soluble in butanol and an extract from aqueous layer. The procedure for the hexane extraction was repeated four times to produce a sufficient amount of hexane-soluble extract. The hexane-soluble, soluble in ethyl acetate, soluble in butanol and water extract were concentrated under reduced pressure to produce the concentrated fractionated hexane extract (0.9406 g dark yellowish brown, yield 6.84% w/w), extract ethyl acetate (2.2900 g dark greenish brown, yield 16.66% w/w), butanol extract (1.0891 g dark reddish brown, 7.92% w/w yield), and water extract (4.2545 g dark brown, yield 30.95% w/w) respectively.

### Standardization of gallic acid

Qualitative HPLC analysis of gallic acid (butanol fraction) in potent fractions was performed by Agilent HPLC system (Series 110) supplied with a quaternary pump, diode array detector (1100 series DAD), automatic sampler, fraction collector and column of biphenyl 150 × 4.6 mM, QC mix 870 (Phenomenex, California, USA) was used. The mobile phase used was a mixture of acetonitrile (90%) and ultrapure water (10%) with a flow rate of 1 mL/minute, at 100 μL/minute of injection (multiple wavelengths; 220, 230, 240, 254, 270, 310, and 360 nm)^45^.

### *Acanthamoeba triangularis*

#### *Acanthamoeba* isolation and cultivation

Water samples were obtained from the Department of Parasitology at the University of Malaya. About 1 mL of filtered water samples were loaded onto non-nutrient agar (NNA) plates. *Escherichia coli* (heat killed), used as a nutrient for the growth of *Acanthamoeba*, was pipetted into the NNA plates and then incubated at 26 °C. Observations were made using an inverted microscope to detect the presence of slow-moving trophozoites with prominent vacuoles and acanthapodia, or cysts with dominant double-walled triangular structures (3–5 arms). Trophozoites were confirmed between 48 to 72 hours after inoculation in the NNA, while cysts were observed after 14 days. The culture of *Acanthamoeba triangularis* (homogenous) was obtained by continuous subcultures before parasite DNA extraction^46^.

#### PCR analysis

*Acanthamoeba triangularis* isolates (trophozoites) were collected from the plate using 5 mL of Page’s Saline. The suspensions were left to centrifuge at 3500 rpm for 10 minutes. Additional DNA extraction was performed using the mini DNA Blood Kit from QIAGEN, Hiden, Germany. Amplification was studied with a PCR mixture containing 25 µL of distilled water, 10X DNA polymerase buffer, 25 mM magnesium chloride, 10 mM deoxynucleotide triphosphate (dNTP) mixture (Thermo Fisher Scientific, Lithuania, USA), 200 moles of each primer: the forward primer JDP1 (5′-GGCCCAGATCGTTACCGTGAA-3′) and the reverse primer JDP2 (5′-TCTCACAAGCTGCTAGGGAGTCA-3′), 1 unit of Taq DNA polymerase (Thermo Fisher Scientific, Lithuania, USA) with 5 µL of DNA template. The process was carried out at 94 °C for 5 minutes and followed by 40 annealing cycles at 84 °C, 60 °C and 72 °C for one minute each and an extension at 72 °C for 5 minutes. The DNA template retrieved with same amount of distilled water was used as a negative control^19,46^.

#### Molecular analysis

PCR amplifications were performed in the 18 S region as a target. PCR amplicon at 450 bp was analysed using electrophoresis gel (1.5%) in Tris-Acetate-EDTA (TAE) buffer. The post-staining protocol was performed using ethidium bromide for visualization under UV light after purification of positive samples that were subsequently sequenced. BLAST software (National Centre for Biotechnology Information) was used for the homology search^46^.

### Preparation of gallic acid (GA) loaded PLGA nanoparticles (Nano-GA- PLGA)

Loading of gallic acid onto nano-PLGA was achieved via the single emulsion method. 0.02 g of PLGA and 0.005 g of gallic acid in 2 mL DCM was vortexed for 1 minute until complete dissolution followed by emulsification by adding drops of this mixture of organic phase in 0.25% aqueous PVA for 10 minutes and then sonicated for 3 minutes at 50% amplitude using a probe sonicator (Athena technology, Maharashtra, India). After removing the DCM by evaporation, the resulting NPs were collected by centrifugation for 5 minutes (10,000 rpm) and washed thrice with distilled water. The resulting nano-GA-PLGA was lyophilized in a freeze dryer, Emitech K750X (Quorum Technologies, Lewes, UK) at −53 °C overnight and stored at −20 °C for further use^33^. The control was prepared following the same method without gallic acid.

### Characterization and surface morphological evaluation of Nano-GA-PLGA

The morphology of nano-GA-PLGA was evaluated by the method previously reported by our group^33^ using scanning electron microscopy (SEM) (FESEM, Quanta FEG 650, FEI, Hillsboro, OR, USA) and transmission electron microscopy (TEM) (Leo Libra-120 (Carl Zeiss AG, Oberkochen, Germany).

### Percent encapsulation and percent release of Nano-GA-PLGA

The gallic acid content in the nano-PLGA was assessed by a spectrophotometer (SpectraMax, M3, Molecular Devices, Washington DC, USA). Free gallic acid was estimated by measuring absorbance at 230 nm^51^ and preparing the standard curve of six known concentrations of gallic acid (0.05 µg/mL to 10 µg/mL). The percentage of gallic acid entrapment was calculated as follows:

$$ \% \,{\rm{gallic}}\,{\rm{acid}}\,{\rm{entrapment}}=\frac{{\boldsymbol{Mass}}\,{\boldsymbol{of}}\,{\boldsymbol{total}}\,{\boldsymbol{drug}}-{\boldsymbol{Mass}}\,{\boldsymbol{of}}\,{\boldsymbol{free}}\,{\boldsymbol{drug}}}{{\boldsymbol{Mass}}\,{\boldsymbol{of}}\,{\boldsymbol{total}}\,{\boldsymbol{drug}}}\ast 100$$

*In vitro* release studies of nano-GA-PLGA were performed in PBS at pH 7.4. An amount of 15 mg of nano-GA-PLGA was added in 40 mL of PBS with consistent stirring for 72 hours at room temperature. At specific time intervals, the sample was collected, and fresh medium was added to maintain the volume. The collected media were centrifuged and the amount of gallic acid released was determined by a UV-visible spectrophotometer at 230 nm^33^.

### Anti-*Acanthamoeba* assay

A 200 µL aliquot of trophozoites suspension or cysts calibrated with the same volume of ethyl acetate, butanol or water fractions of *L. indica* (0.5 mg/mL, 1.0 mg/mL and 1.5 mg/mL), gallic acid and nano-GA-PLGA at various concentrations such as 25 (146.9 µM), 50 (293.9 µM) and 100 µg/mL (587.8 µM) were carefully mixed in microcentrifuge tubes. Then, the tubes were kept in a standard incubator at 26 °C in the dark for 24, 48 and 72 hours. The similar procedure was applied to control tubes containing only 200 µL of sterile distilled water with 200 µL of trophozoite/cyst suspension. Chlorhexidine, a member of biguanide, used in the treatment of amoebic keratitis, was used as a reference drug control (positive control) and subjected to the same procedure. Chlorhexidine was used in concentrations of 4 µg and 25 µg in 1 mL of sterile distilled water for trophozoites and cysts, respectively^52,53^. After the incubation period of 24, 48 and 72 hours at 26 °C, 10 µL of trophozoite or cyst suspension were transferred to 10 µL of 0.4% trypan blue. Unstained (viable) and stained (non-viable) trophozoites/cysts were individually counted in the hemocytometer counting chamber, 3 minutes after stain addition. Unstained/stained trophozoites or cysts were counted and listed^46^.

### Cytotoxicity tests

The cytotoxicity assay of nano-GA-PLGA was used to determine the cytotoxicity profile of gallic acid, PLGA NPs (without compound) and nano-GA-PLGA in MRC-5 cell lines (lung epithelial cells). Six different concentrations of each sample ranging from 10 µg/mL to 120 µg/mL were investigated using the MTT assay, as previously described by Mosmann (1983). MRC-5 cells were cultured in complete growth medium (Roswell Park Memorial Institute (RPMI) with 10% Fetal Bovine Serum (FBS)). Five to six passages were made. Cultures were further checked and media changed after 48–72 hours, depending on growth. At each passage, cells were seeded in 25 cm^3^ flasks, (Nunc, Roskilde, Denmark) with a density of 2.5 × 10^5^ cells/mL. Cells were seeded in 96-well plates at a density of 10^6^ cells/well. To allow cells to adhere to well, cells were incubated at 37 °C in a standard CO_2_ incubator with 5% CO_2_ for 24 hours before the addition of the gallic acid and synthesized nanoparticles. Then, cells were exposed to treatment for additional 24, 48 and 72 hours after the addition of gallic acid and prepared nanoparticles. In MTT assays, purple formazan crystals are formed by reducing the yellow tetrazolium salt caused by respiratory cells (living cells). The spectrophotometric absorbance was measured at 570 nm, using SpectraMax (M3, Molecular Devices, Washington DC, USA). Cells without treatments with the test reagents were taken as untreated cells with 100% viability, whereby cells with only the RPMI 1640 media were used as a blank^45^.

The percentage of cell viability was calculated using the formula:

$$ \% \,{\rm{viability}}=\frac{{\bf{A}}{\bf{b}}{\bf{s}}({\bf{s}}{\bf{a}}{\bf{m}}{\bf{p}}{\bf{l}}{\bf{e}})-{\bf{A}}{\bf{b}}{\bf{s}}({\bf{b}}{\bf{l}}{\bf{a}}{\bf{n}}{\bf{k}})}{{\bf{A}}{\bf{b}}{\bf{s}}({\bf{u}}{\bf{n}}{\bf{t}}{\bf{r}}{\bf{e}}{\bf{a}}{\bf{t}}{\bf{e}}{\bf{d}})-{\bf{A}}{\bf{b}}{\bf{s}}({\bf{b}}{\bf{l}}{\bf{a}}{\bf{n}}{\bf{k}})}\times 100$$

### Apoptotic effect of Lee indica fractions, GA and Nano-GA-PLGA

The apoptotic effect of *Lee indica* fractions, GA and nano-GA-PLGAs was determined using the terminal deoxynucleotidyltransferase-mediated dUTP-biotin nick end labelling (TUNEL) apoptotic detection kit (S7100, Millipore, Burlington, MA, USA). The apoptotic detection kit was used according to the manufacturer’s protocol. In brief, the experiment was performed in a 6 well plate. The stages of trophozoites of *Acanthamoeba triangularis* were cultured at a concentration of 10^6^ cells/mL. Parasites were treated with various concentrations of *Lee indica* fractions, GA and nano-GA-PLGA, which were incubated for 24 hours. Cells without plant extracts, gallic acid and GA-PLGA nanoparticles were used as controls. After 24 hours of incubation period, cells were collected and fixed on silanized slides with 1% paraformaldehyde before the TUNEL assay. This assay is based on the binding of terminal deoxynucleotidyl transferase (TdT) and the conjugated drug and peroxidase, converting the substrate, a mixture of hydrogen peroxide and 3’3- diaminobenzidine (DAB) into a brown precipitate. Hence, apoptotic cells form a brown precipitate, detected under a light microscope^33^.

### Statistical analysis

Data were entered, edited and analyzed using SPSS (Statistical Package for Social Sciences) software version 21 (SPSS, Chicago, IL, USA). Results were presented as mean ± standard deviation of independent experiments in triplicate. The means were analyzed by Student’s unpaired t-test to estimate the difference between the test sample and the negative and positive control (Chlorhexidine). The difference was considered significant when the p-value was below 0.05 for the student’s unpaired t-test.

## Conclusions

In conclusion, our results indicate that the medicinal plant *Leea indica* and its fractions may provide a new therapeutic agent against infections caused by free living amoebae. The butanol fraction of *L. indica* is able to induce death in *Acanthamoeba* cells by apoptotic pathway. Gallic acid, a strong antioxidant, resulted in less cytotoxic agent after being encapsulated in PLGA nanoparticles. Hence, nanotechnology can be used as promising new drug delivery system in order to reduce the cytotoxic effect of natural compounds.

## Patents

A patent on amoebicidal activity of *Leea indica* has been filed at The University of Malaya Centre of Innovation and Commercialization (UMCIC), University of Malaya, Malaysia with reference number: SK/P1611/UM/18, 09/07/2018.

## Supplementary information

Supplementary Figures.
