# Supplementary material for: PLGA nanoparticles loaded with Gallic acid- a constituent of Leea indica against Acanthamoeba triangularis
Source: Sci Rep. 2020 Jun 2;10:8954. doi: 10.1038/s41598-020-65728-0 (PMC7265533; doi:10.1038/s41598-020-65728-0)
Supplement: Supplementary file 1 — Supplementary Figures. [file 41598_2020_65728_MOESM1_ESM.pdf]

Supporting Information

PLGA nanoparticles loaded with Gallic acid- a constituent of *Leea indica* against *Acanthamoeba triangularis*

Tooba Mahboob[1,2], Muhammad Nawaz[3*], Maria de Lourdes Pereira[4], Tan Tian-Chye[2], Chandramathi Samudi[1], Shamala Devi Sekaran[5], Christophe Wiart[6] and Veeranoot Nissapatorn[2,7,8*]

[1]Department of Medical Microbiology, Faculty of Medicine, University of Malaya, 50603 Kuala Lumpur, Malaysia; tooba666@hotmail.com, chandramathi@um.edu.my

[2]Department of Parasitology, Faculty of Medicine, University of Malaya, 50603 Kuala Lumpur, Malaysia; tantianchye@um.edu.my

[3]Department of Nano-Medicine Research, Institute for Research and Medical Consultations (IRMC), Imam Abdulrahman Bin Faisal University, P.0. Box 1982, 31441 Dammam, Saudi Arabia; mnnmuhammad@iau.edu.sa, nawwaz@gmail.com

[4]Department of Medical Sciences & CICECO-Aveiro Institute of Materials, University of Aveiro, 3810-193 Aveiro, Portugal; mlourdespereira@ua.pt

[5]Faculty of Medicine, MAHSA University, Saujana Putra Campus, Malaysia; shamalamy@yahoo.com

[6]School of Pharmacy, Nottingham University Malaysia Campus, 43500 Semenyih, Selangor, Kuala Lumpur, Malaysia; Christophe.Wiart@nottingham.edu.my

[7]School of Allied Health Sciences (World Union for Herbal Drug Discovery), Walailak University, 80161 Nakhon Si Thammarat, Thailand

[8]Research Excellence Center for Innovation and Health Products (RECIHP), Walailak University, 80161 Nakhon Si Thammarat, Thailand; nissapat@gmail.com

***Correspondence to**:

Veeranoot Nissapatorn

veeranoot.ni@wu.ac.th, nissapat@gmail.com;

Muhammad Nawaz

mnnmuhammad@iau.edu.sa, nawwaz@gmail.com

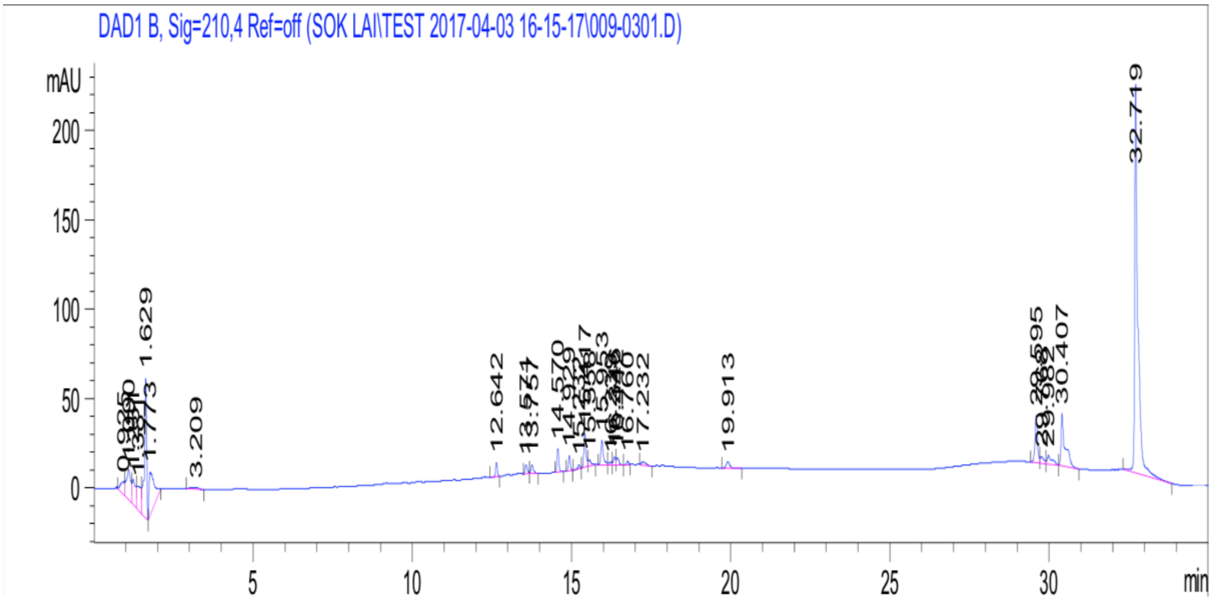

Figure S1: HPLC Analysis of Gallic acid Standard

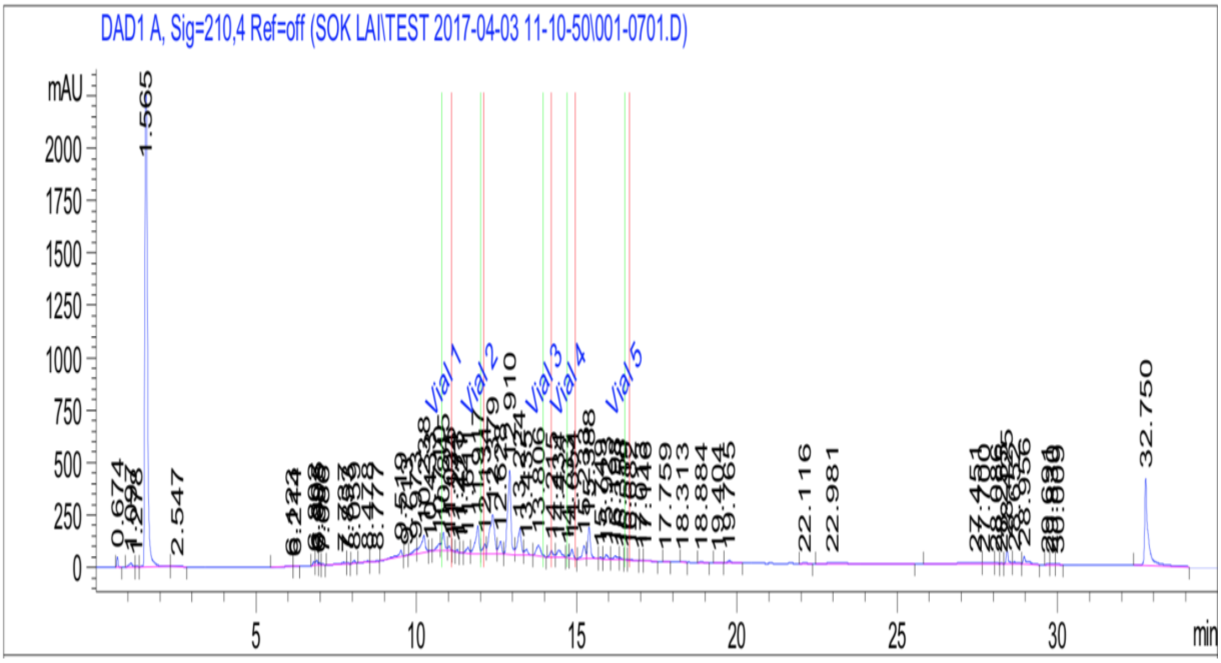

Figure S2: HPLC Analysis of *Leea indica* butanol Extract

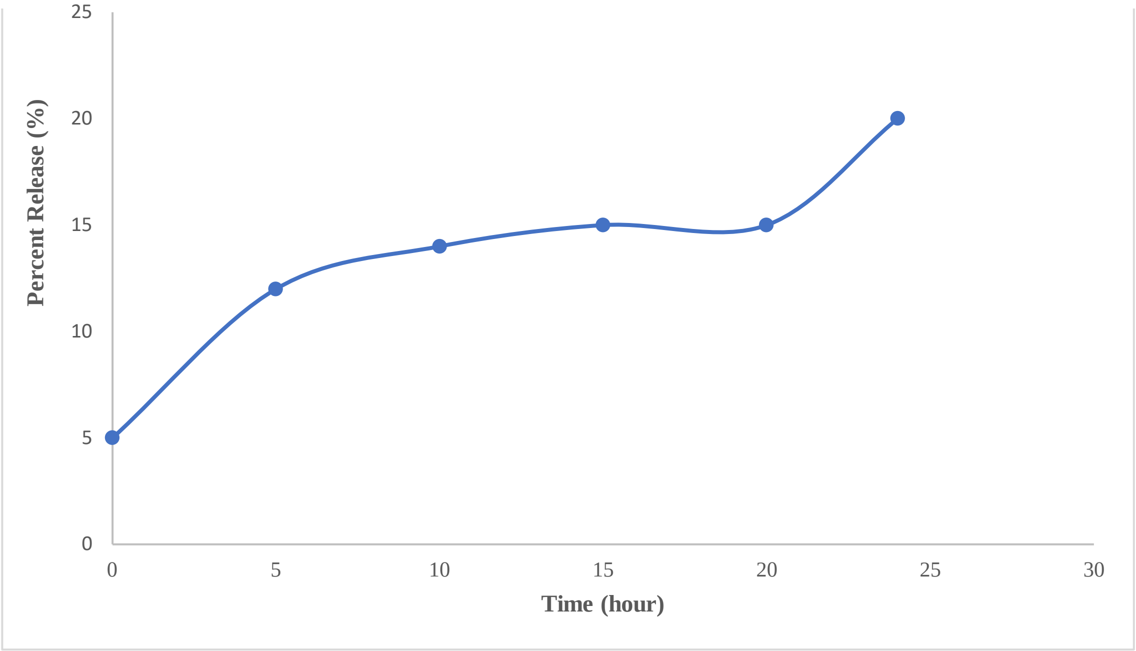

Figure S3: Percentage release of Nano-GA-PLGA within 24 hours

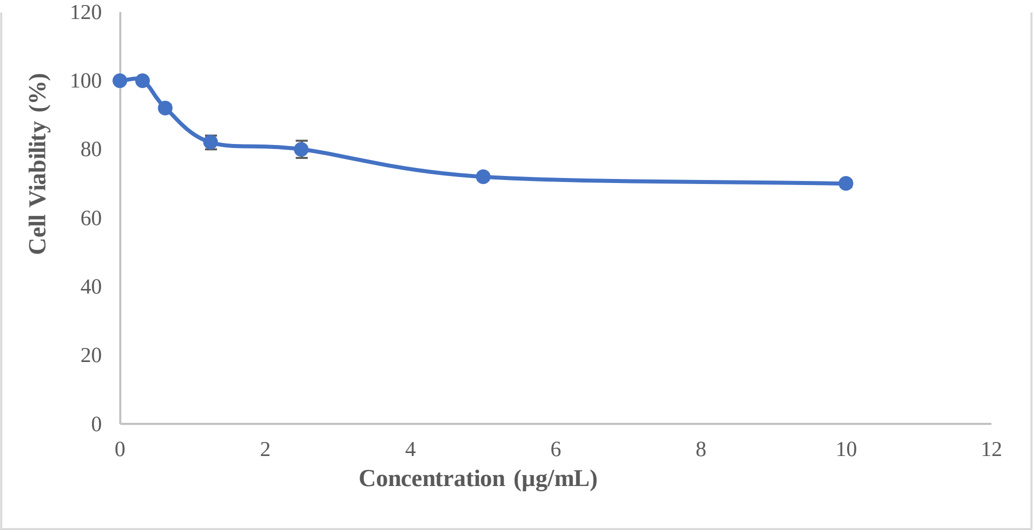

Figure S4: Cytotoxicity Test of Chlorhexidine against Lung epithelial cell line (MRC-5)
